# Endoscopic management of bile leaks following T-tube removal in liver transplant recipients with end-to-end anastomosis using fully covered self-expandable metal stents

**DOI:** 10.1093/jscr/rjag630

**Published:** 2026-07-24

**Authors:** Songming Ding, Yang Liu, Shanjie Dong, Yiting Hu, Hengkai Zhu, Shusen Zheng, Qiyong Li

**Affiliations:** Division of Hepatobiliary and Pancreatic Surgery, Shulan (Hangzhou) Hospital Affiliated to Zhejiang Shuren University, Shulan International Medical College, No. 848 Dongxin Road, Hangzhou, 310003, P.R. China; Endoscopy Center, Shulan (Hangzhou) Hospital Affiliated to Zhejiang Shuren University, Shulan International Medical College, No. 848 Dongxin Road, Hangzhou, 310003, P.R. China; Division of Hepatobiliary and Pancreatic Surgery, Shulan (Hangzhou) Hospital Affiliated to Zhejiang Shuren University, Shulan International Medical College, No. 848 Dongxin Road, Hangzhou, 310003, P.R. China; Division of Hepatobiliary and Pancreatic Surgery, Shulan (Hangzhou) Hospital Affiliated to Zhejiang Shuren University, Shulan International Medical College, No. 848 Dongxin Road, Hangzhou, 310003, P.R. China; Division of Hepatobiliary and Pancreatic Surgery, Shulan (Hangzhou) Hospital Affiliated to Zhejiang Shuren University, Shulan International Medical College, No. 848 Dongxin Road, Hangzhou, 310003, P.R. China; Division of Hepatobiliary and Pancreatic Surgery, Shulan (Hangzhou) Hospital Affiliated to Zhejiang Shuren University, Shulan International Medical College, No. 848 Dongxin Road, Hangzhou, 310003, P.R. China; Division of Hepatobiliary and Pancreatic Surgery, Shulan (Hangzhou) Hospital Affiliated to Zhejiang Shuren University, Shulan International Medical College, No. 848 Dongxin Road, Hangzhou, 310003, P.R. China

**Keywords:** bile leak, T-tube, liver transplantation, endoscopic retrograde cholangiopancreatography, fully covered self-expandable metal stent

## Abstract

Bile leaks (BLs) following T-tube removal, while typically not life-threatening, can result in serious complications if diagnosis or treatment is delayed. Endoscopic retrograde cholangiopancreatography (ERCP) is currently the preferred modality for managing such leaks in liver transplantation (LT) recipients. We present a retrospective case series of six LT patients who developed BLs after T-tube removal and were successfully managed using fully covered self-expandable metal stents (FCSEMSs). This report details our clinical experience alongside a review of the existing literature regarding this specific clinical entity.

## Introduction

Despite advances in surgical techniques, biliary complications (BCs) remain the “Achilles’ heel” of liver transplantation (LT), contributing substantially to postoperative morbidity. The incidence of BCs ranges from 5% to 25% following whole-organ transplantation, with associated mortality rates reaching up to 10% [1]. Among these, bile leaks (BLs) represent the most frequent cause of biliary tract-related mortality [2, 3]. These leaks may originate at the anastomotic site; from the cystic duct stump or Luschka ducts; from the cut surface of the graft; or following T-tube removal [4]. Endoscopic retrograde cholangiopancreatography (ERCP) is established as the first-line therapeutic modality for BLs post-LT, demonstrating a success rate of 92.8% (65/69) [5].

This study reports on six patients who developed BLs following T-tube removal after undergoing LT with end-to-end anastomosis and were subsequently treated with fully covered self-expandable metal stent (FCSEMS) placement. By detailing the clinical data and outcomes alongside a review of the literature, we aim to enhance the understanding of this potential complication associated with T-tube usage.

## Case 1

**Figure 1:**
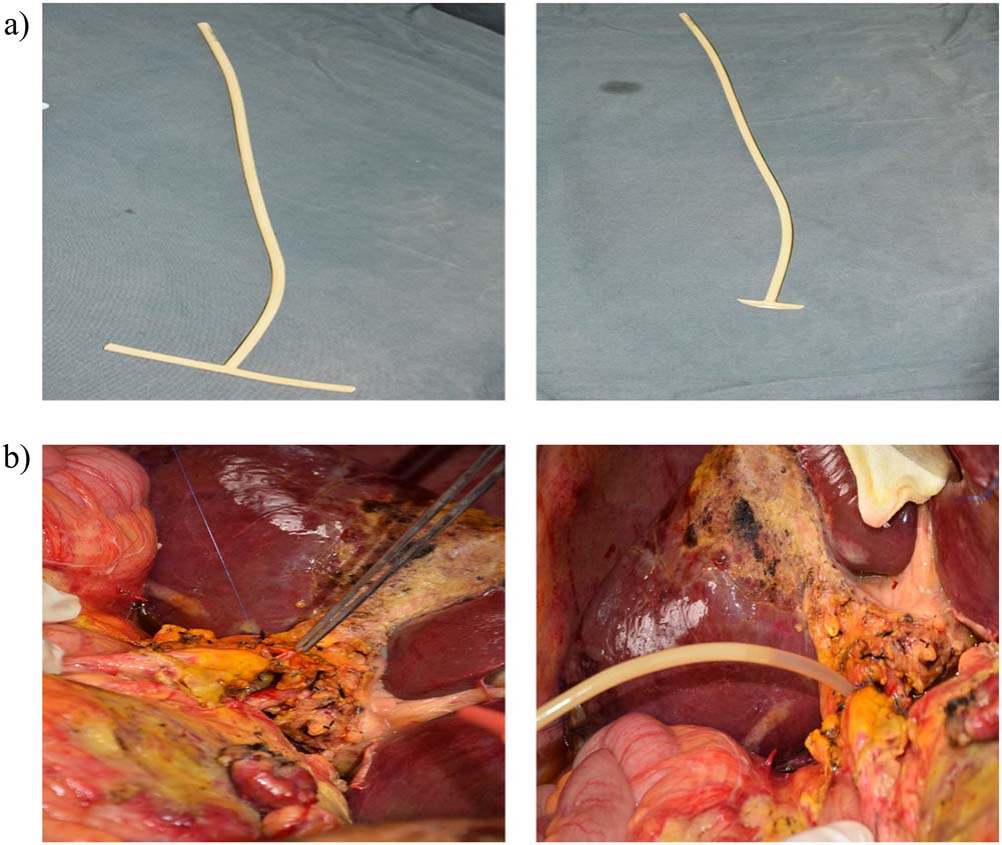
T-tube shape and insertion method. (a) T-tube shape (b) T-tube insertion site and suture method.

A 51-year-old female underwent ABO blood group (ABO)-incompatible LT for hepatic coma secondary to hepatitis B-related cirrhosis and liver failure. During the biliary reconstruction phase of the operation, the T-tube was trimmed as depicted in Fig. 1a. The surgical technique involved suturing the posterior wall of the bile duct first, followed by insertion of the T-tube into the anastomotic site. One limb of the T-tube was positioned within the donor bile duct, and the other within the recipient bile duct. Subsequently, interrupted sutures were placed to complete the anastomosis of the anterior bile duct wall (Fig. 1b).

**Figure 2:**
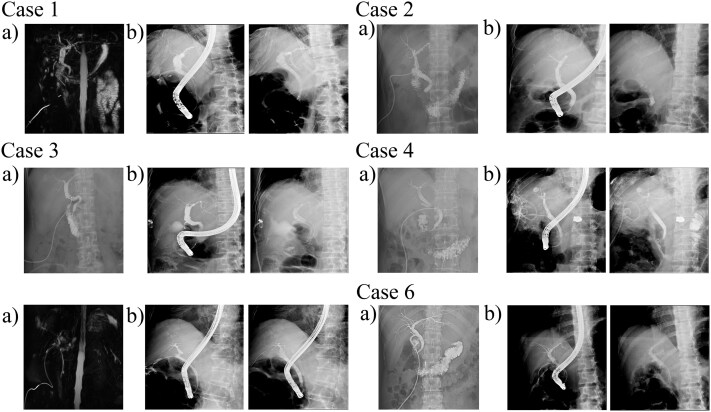
Pre-T-tube removal imaging via T-tube cholangiography or magnetic resonance cholangiopancreatography (a), and specific ERCP treatment (b).

During the recovery period, she experienced transaminase elevations, and a liver biopsy confirmed antibody-mediated rejection. Eight months post-LT, immediately following T-tube removal, she developed abdominal pain, fever, nausea, and vomiting. Percutaneous drainage yielded bilious fluid with a maximum daily output of 970 ml. Serum total bilirubin (TB) rose from 14 μmol/l before T-tube removal to 34 μmol/l pre-ERCP. ERCP confirmed a BL, and an FCSEMS (10 × 8 cm) was placed without sphincterotomy (Fig. 2). The leak resolved successfully, and the patient was discharged 17 days post-ERCP. The stent was removed 8 months later.

## Case 2

A 57-year-old male underwent ABO-compatible LT for acute liver failure. During the recovery period, transaminase levels rebounded, and liver biopsy pathology showed mild chronic liver injury with mild cholestasis. Approximately 7 months post-LT, following T-tube removal, the patient developed abdominal pain, fever, and diarrhea. Percutaneous drainage confirmed a BL, with a maximum daily bilious output of 1150 ml. Serum TB rose from 12 μmol/l before T-tube removal to 32 μmol/l pre-ERCP. ERCP with sphincterotomy was performed, utilizing an FCSEMS (10 × 6 cm) to seal the leak (Fig. 2). Twelve days post-ERCP, the patient was discharged. The FCSEMS was removed 7 months later.

## Case 3

A 54-year-old female underwent ABO-compatible LT for hepatitis B-related cirrhosis and liver failure. Six months post-LT, following T-tube removal, the patient developed abdominal pain, fever, nausea, vomiting, and abdominal distension. Percutaneous drainage confirmed a BL, with a maximum daily output of 450 ml. An FCSEMS (8 × 8 cm) was placed via ERCP with sphincterotomy (Fig. 2). The patient recovered well and was discharged 5 days post-ERCP. At the 3-month follow-up, the metal stent was found to have spontaneously passed.

## Case 4

A 59-year-old male underwent LT for hepatocellular carcinoma (HCC). Seven months post-LT, following T-tube removal, the patient developed abdominal pain, distension, and chest tightness. At the time of T-tube removal, bilateral lung metastases, sternal metastases, and peritoneal metastases in the pelvic floor were already present. The pulmonary metastases received intensity-modulated radiation therapy. However, the tumor did not invade the bile duct, and there was no tumor thrombus within it. Thoracic drainage indicated a BL; thoracentesis revealed bilious fluid with a maximum daily output of 1200 ml. ERCP with sphincterotomy and FCSEMS placement (8 × 6 cm) was performed (Fig. 2). The patient was discharged 7 days later. The FCSEMS was removed 6 months post-procedure. The patient succumbed to disease recurrence 19 months post-transplant.

## Case 5

A 54-year-old female underwent ABO-compatible LT for autoimmune hepatitis and cirrhosis. During the recovery period, liver biopsy showed mild cholestatic liver injury. Ten months post-LT, the T-tube was removed. Subsequently, the patient developed abdominal pain, fever, nausea, vomiting, and abdominal distension. Serum TB rose from 20 μmol/l before T-tube removal to 71 μmol/l pre-ERCP. An ERCP with sphincterotomy and FCSEMS placement (8 × 8 cm) was performed (Fig. 2). The patient was discharged 6 days post-ERCP. The FCSEMS was removed 6 months later.

## Case 6

A 41-year-old female underwent ABO-incompatible LT for hepatitis B-related liver failure. Four months post-LT, following T-tube removal, she developed abdominal pain, fever, nausea, and vomiting. Abdominal paracentesis drained a maximum of 800 ml of bilious fluid daily. Subsequently, an FCSEMS (8 × 6 cm) was inserted via ERCP with sphincterotomy (Fig. 2). Ten days post-ERCP, the patient was discharged with resolved symptoms. At the 4-month follow-up, the metal stent was found to have spontaneously passed.

## Discussion

The use of T-tubes in LT with end-to-end anastomosis has been debated for decades. Some have concluded that the use of a T-tube during LT is not justified due to the increased risk of BLs, biliary infections, and even early allograft dysfunction [6–8]. Conversely, others report that T-tube placement during LT is a prudent strategy for patients at high risk of biliary strictures (BSs) [9–11], noting that BCs in these cases tend to be less severe [12].

Undoubtedly, the overwhelming mismatch between the demand for LT and the shortage of donor organs represents a silent crisis. The use of marginal donor grafts, particularly livers procured from donors after circulatory death, has expanded the liver supply pool to alleviate this critical situation. Consequently, the utility of T-tubes in LT warrants renewed discussion [13], and transplant centers in Portugal provided specific indications for their use [14].

Techniques for T-tube insertion and removal protocols are continuously evolving and remain non-uniform. Literature reports indicate that using 5F pediatric T-tubes with refined insertion and removal protocols can reduce the incidence of biliary peritonitis following T-tube removal to 4%, compared to the 5%–33% incidence reported in a systematic review [13]. While the optimal timing for T-tube removal remains uncertain, it is generally recommended that the tube be retained for at least 3–6 months after LT [8]. Studies have shown that removal earlier than 6 months in deceased donor LT is a risk factor for BS, whereas removal earlier than 8 months in living donor LT is a risk factor for BL [15].

In this case series, the mean T-tube indwelling time was 7 months. Clinical manifestations of BLs included abdominal pain (6/6), fever (5/6), nausea/vomiting (4/6), abdominal distension (3/6), and diarrhea (1/6). One patient developed a pleural fistula with large effusion, potentially associated with prior lung radiotherapy. Elevated serum TB levels were observed in three patients. The technical success rate of ERCP was 100% (6/6), aligning with the 92.8% benchmark in existing literature [5]. Diverging from studies advocating endoscopic nasobiliary drainage (ENBD) [5], our protocol prioritized FCSEMS (8–10 mm), especially for anastomotic narrowing or tortuosity. Of the six FCSEMS placements, four were successfully explanted, while two underwent spontaneous migration.

As reported in the literature, percutaneous puncture drainage is necessary when imaging examination reveals frank fluid collection [16]. This can alleviate patients’ anxiety and avoid the formation of bilomas, as well as reduce the impact of bile on liver blood flow. Surgical repair is recommended for patients with recurrent or persistent BLs after endoscopic treatment [5].

## Conclusion

BL following T-tube removal is a challenging but manageable complication in LT recipients. Our experience demonstrates that ERCP with the placement of FCSEMS is a highly effective and safe therapeutic strategy for sealing such leaks.

## References

[1] Bofill A, Cárdenas A. A practical approach to the endoscopic management of biliary strictures after liver transplantation. Ann Hepatol 2024;29:101186. 10.1016/j.aohep.2023.101186 38035999

[2] Oh D, Lee SK, Song TJ et al. Endoscopic management of bile leakage after liver transplantation. Gut Liver 2015;9:417–23. 10.5009/gnl14117 25717048 PMC4413977

[3] Magro B, Tacelli M, Mazzola A et al. Biliary complications after liver transplantation: current perspectives and future strategies. HepatoBiliary Surg Nutr 2021;10:76–92. 10.21037/hbsn.2019.09.01 33575291 PMC7867735

[4] Macías-Gómez C, Dumonceau JM. Endoscopic management of biliary complications after liver transplantation: an evidence-based review. World J Gastrointest Endosc 2015;7:606–16. 10.4253/wjge.v7.i6.606 26078829 PMC4461935

[5] Saab S, Martin P, Soliman GY et al. Endoscopic management of biliary leaks after T-tube removal in liver transplant recipients: Nasobiliary drainage versus biliary stenting. Liver Transpl 2000;6:627–32. 10.1053/jlts.2000.8200 10980063

[6] Kalisvaart M, de Jonge J, Abt P et al. The role of T-tubes and abdominal drains on short-term outcomes in liver transplantation-a systematic review of the literature and expert panel recommendations. Clin Transplant 2022;36:e14719. 10.1111/ctr.14719 35596705 PMC10078006

[7] Sotiropoulos GC, Sgourakis G, Radtke A et al. Orthotopic liver transplantation: T-tube or not T-tube? Systematic review and meta-analysis of results. Transplantation 2009;87:1672–80. 10.1097/TP.0b013e3181a5cf3f 19502959

[8] Pravisani R, de Simone P, Patrono D et al. An Italian survey on the use of T-tube in liver transplantation: old habits die hard! Updates Surg 2021;73:1381–9. 10.1007/s13304-021-01019-1 33792888 PMC8397659

[9] López-Andújar R, Orón EM, Carregnato AF et al. T-tube or no T-tube in cadaveric orthotopic liver transplantation: the eternal dilemma: results of a prospective and randomized clinical trial. Ann Surg 2013;258:21–9. 10.1097/SLA.0b013e318286e0a0 23426348

[10] Song SM, Lu TT, Yang WW et al. T-tube or no T-tube for biliary tract reconstruction in orthotopic liver transplantation: an updated systematic review and meta-analysis. Expert Rev Gastroenterol Hepatol 2021;15:1201–13. 10.1080/17474124.2021.1903874 33720798

[11] Riediger C, MM Mül, Michalski CW et al. T-tube or No T-tube in the reconstruction of the biliary tract during orthotopic liver transplantation: systematic review and meta-analysis. Liver Transpl 2010;16:705–17. 10.1002/lt.22070 20517904

[12] Weiss S, Schmidt SC, Ulrich F et al. Biliary reconstruction using a side-to-side choledochocholedochostomy with or without T-tube in deceased donor liver transplantation: a prospective randomized trial. Ann Surg 2009;250:766–71. 10.1097/SLA.0b013e3181bd920a 19809299

[13] Spoletini G, Bianco G, Franco A et al. Pediatric T-tube in adult liver transplantation: technical refinements of insertion and removal. World J Gastrointest Surg 2021;13:1628–37. 10.4240/wjgs.v13.i12.1628 35070068 PMC8727192

[14] Carmelino J, Rodrigues S, Marques HP et al. Biliary anastomosis in liver transplantation: with or without T-tube? Acta Med Port 2017;30:122–6. 10.20344/amp.7287 28527479

[15] Wang SH, Lin PY, Wang JY et al. Predictors of biliary leakage after T-tube removal in living donor liver transplantation recipients. Transplant Proc 2015;47:2488–92. 10.1016/j.transproceed.2015.09.019 26518957

[16] Moy BT, Birk JW. A review on the management of biliary complications after orthotopic liver transplantation. J Clin Transl Hepatol 2019;7:61–71. 10.14218/JCTH.2018.00028 30944822 PMC6441650

